# *Arabidopsis thaliana* detoxification gene *AtDTX1* is involved in trichothecene 3-acetyl-deoxynivalenol efflux

**DOI:** 10.3389/fpls.2025.1574367

**Published:** 2025-05-19

**Authors:** Guixia Hao, Jackson Edwards, Nicholas Rhoades, Susan P. McCormick

**Affiliations:** ^1^ Mycotoxin Prevention and Applied Microbiology Research Unit, National Center for Agricultural Utilization Research, United States Department of Agriculture (USDA), Agricultural Research Service, Peoria, IL, United States; ^2^ Mycotoxin Prevention and Applied Microbiology Research Unit, Oak Ridge Institute for Science and Education, National Center for Agricultural Utilization Research, United States Department of Agriculture (USDA), Agricultural Research Service, Peoria, IL, United States

**Keywords:** *Fusarium graminearum*, trichothecene, 3-acetyl-deoxynivalenol, *Arabidopsis thaliana* transporter, efflux

## Abstract

The fungal pathogen *Fusarium graminearum* causes Fusarium head blight (FHB) on wheat and produces trichothecene mycotoxins that contaminate grains. Deoxynivalenol (DON) and its acetylated derivatives, including 3-acetyl-DON (3-ADON) and 15-acetyl-DON (15-ADON), are the most common trichothecenes found in contaminated grains, which causes food and feed safety issues. Approaches that detoxify DON can reduce FHB and mycotoxin contamination. Our previous study showed that transgenic *Arabidopsis thaliana* expressing a *F. graminearum* 3-O-acetyltransferase self-protection enzyme (FgTri101), converted DON to 3-ADON and excreted 3-ADON out of plant cells to protect plant growth and development. The goal of the current study was to identify the transporter involved in 3-ADON excretion and utilize it to reduce toxicity and FHB. To identify trichothecene transporter candidates, transcriptomic studies were conducted on *FgTri101* transgenic *A. thaliana* seedlings treated with DON (50 mg/L, 24 h) versus untreated controls. Transcriptomic analyses revealed that three transporter genes, including two *A. thaliana* detoxification genes (*AtDTX1* and *AtDTX3*) and one ABC transporter (*ABCB4*), were upregulated by DON treatment. *Atdtx1* mutant transported 3-ADON less efficiently than *Atdtx3* and *Atabcb4* mutants. Therefore, the *A. thaliana* Col-0 mutant *Atdtx1* line was transformed and expressed *FgTRI101*. The *Atdtx1* mutant lines expressing *FgTRI101* showed resistance to DON but had significantly shorter roots than the *FgTRI101* Col-0 transgenic line. Furthermore, significantly less 3-ADON was detected in the media used to grow the transgenic *Atdtx1* mutant seedlings expressing *FgTRI101* than the Col-0 seedlings expressing *FgTRI101*. These data indicate that *AtDTX1* is involved in efflux of 3-ADON and that at least another transporter or other mechanism is associated with 3-ADON transport.

## Introduction

Trichothecenes are toxic sesquiterpenoid metabolites produced by some *Fusarium* species. Trichothecenes harm animal and human health by inhibiting eukaryotic cell functions, including DNA and RNA synthesis, protein translation and elongation, mitochondrial translation, and cell division (Rocha et al., 2005; Bin-Umer et al., 2011). *F. graminearum* is a primary causal agent of Fusarium head blight (FHB), a disease of wheat, barley and other cereals that contaminates grains with harmful mycotoxins. The common trichothecene mycotoxins found in contaminated cereal grains include deoxynivalenol (DON), 3-acetyl-DON (3-ADON), 15-acetyl-DON (15-ADON), nivalenol (NIV) and T-2 toxin (Foroud et al., 2019). DON inhibits protein synthesis by interacting with the 60S peptidyl transferase of eukaryotic ribosomes (Garreau de Loubresse et al., 2014). While DON, 3-ADON and 15-ADON exhibit similar toxicological effects on humans and animals, these trichothecene analogs show different levels of toxicity to plants. DON can cause plant cell death, reactive oxygen responses, and plant defense gene activation (Desmond et al., 2008). In addition, DON acts as a virulence factor in wheat, promoting disease development and causing premature bleaching of wheat heads, a typical FHB symptom (Proctor et al., 1995; Bai et al., 2002). A recent study revealed that DON facilitates the traversal of the cell wall through plasmodesmata during *F. graminearum* colonization of wheat tissues (Armer et al., 2024). Compared to DON, 3-ADON is less toxic to plants. For example, transgenic Arabidopsis and rice expressing *F. graminearum TRI101*, encoding a 3-O-acetyltransferase that converts DON to 3-ADON, have increased plant resistance to the toxin (Ohsato et al., 2007; Hao et al., 2021). Transgenic wheat expressing *F. graminearum TRI101* have enhanced FHB resistance and reduced mycotoxin contamination (Yulfo-Soto et al., 2024). In response to mycotoxins produced by fungi during plant infection, plants deploy toxin detoxification strategies for self-protection. Multiple plant detoxification genes have been identified and utilized to reduce toxicity and enhance disease resistance, including plant UDP-glycosyltransferases (UGTs) (Poppenberger et al., 2003; Li et al., 2015) and glutathione transferases (GSTs) that convert DON to less toxic derivatives (Wang et al., 2020).

In addition to detoxifying toxic compounds, plants employ transporters to sequester or dispose of diverse secondary metabolites, including toxins. Several major types of transporters have been described, including the ATP-binding cassette (ABC) family, the major facilitator superfamily (MFS), the resistance-nodulation-division (RND) family, the small multidrug resistance (SMR) transporters, and multidrug and toxic compound extrusion (MATE) protein family (also called Detoxification Efflux Carriers, DTXs). A typical DTX protein contains 440–550 amino acids and a conserved transmembrane domain comprising 12 alpha-helices. Many DTX transporters have been characterized that transport toxic compounds or other secondary metabolites in plant species (Chen et al., 2020). For instance, *Nicotiana tabacum* NtJAT1 has been shown to transport alkaloids from the cytosol to the vacuole, thereby regulating plant development and disease resistance (Morita et al., 2009). In the *A. thaliana* genome, 56 DTXs are predicted, and some have been functionally characterized. *A. thaliana AtDTX1* serves as an efflux carrier for antibiotics, toxic compounds, heavy metals and plant alkaloids (Li et al., 2002). The *A. thaliana AtDTX41* transporter, identified in transparent testa12 mutants, moves proanthocyanidin precursors into the vacuole (Marinova et al., 2007). *A. thaliana AtDTX35* is a flavonoid transporter that is essential for anther dehiscence and pollen development (Thompson et al., 2010). Both *AtDTX35* and transporter *AtDTX33* act as chloride channels for turgor regulation during root-hair elongation and stomatal movement (Zhang et al., 2017). During plant-fungal pathogen interactions, *A. thaliana AtDTX18* exports hydroxycinnamic acid amides to the leaf surface where they inhibit *Phytophthora infestans* spore germination (Dobritzsch et al., 2016).

In addition to DTXs, ABC transporters utilize the energy of ATP hydrolysis to actively transport many chemically and structurally unrelated compounds across cellular membranes. Many toxic secondary metabolites are conjugated and moved by ABC transporters to the plant vacuole for sequestration. In the *A. thaliana* genome, a total of 130 annotated ABC transporters are predicted, but thus far only about 20 of them have been functionally characterized (Kang et al., 2011). For example, *AtMRP1* and *AtMRP2*, have been shown to transport glutathione-conjugates and chlorophyll catabolites to vacuoles for sequestration (Lu et al., 1997, 1998). Although multiple *A. thaliana* transporters have been functionally characterized, none of them are known to be involved in mycotoxin transport. Identification and characterization of mycotoxin transporters may provide novel targets for reducing mycotoxin contamination and improving food safety.

Our previous study showed that transgenic A. thaliana expressing a F. graminearum self-protection gene, FgTRI101, converted the mycotoxin DON to 3-ADON and excreted 3-ADON out of plant cells to protect plant growth and development (Hao et al., 2021). The finding of 3-ADON excretion into media suggests that *A. thaliana* has a transporter for 3-ADON efflux. Furthermore, our recent study showed that transgenic wheat expressing *FgTRI101* has increased FHB resistance and reduced DON contamination (Yulfo-Soto et al., 2024). Therefore, identification of a 3-ADON transporter and expression of it in wheat could be used to increase 3-ADON disposal and improve resistance to FHB. To identify trichothecene transporters in *A. thaliana*, in the current study, we performed transcriptomic studies using FgTri101 transgenic *A. thaliana* challenged with DON. The *AtDTX1* gene was identified and was further characterized for its role on 3-ADON efflux in *A. thaliana* using *A. thaliana* mutants expressing *FgTRI101.*

## Materials and methods

### Chemical preparation and sample treatments

DON and 3-ADON were produced and purified at the Mycotoxin Prevention and Applied Microbiology Research Unit, USDA/ARS, Peoria, IL as described previously (Desjardins et al., 2007). DON was dissolved in sterilized water at 2 mg/mL as a stock. 3-ADON (10 mg) was dissolved in 100 uL methanol and brought up to 2 mg/mL with sterilized water.

Homozygous *A. thaliana* T_2_ seedlings expressing *FgTRI101* were treated with 50 mg/L DON for 24 h in RNA-seq experiments because our previous study showed that the converted 3-ADON was present at 24 or 48 h in liquid medium used to culture *FgTRI101* transgenic seedlings (Hao et al., 2021). Briefly, the *FgTRI101* transgenic seeds were surface disinfected using 70% ethanol for 2 min, then 10% Clorox for 10 min, and rinsed three times with sterile water. The sterilized seeds were placed on Murashige and Skoog (MS) agar plates containing 50 mg/L kanamycin. Subsequently, the plates were kept at 4°C for 2 days and transferred to a growth chamber at 23/20°C with 14 h light/10 h-dark cycles (150 µmol/ms light intensity). After 9 to 10 days, 16–18 seedlings were transferred to a 50 mL tube containing 5 mL of half-strength MS liquid medium with 1% sucrose. After 3 to 4 days, the medium was removed and substituted with 5 mL of fresh medium supplemented with 50 mg/L DON or without DON (as controls). Three biological replicates were conducted for each treatment. The seedlings were collected at 24 h following DON treatment for RNA isolation. DON and 3-ADON were extracted from the liquid medium and measured to confirm DON to 3-ADON conversion and 3-ADON excretion.

### RNA-seq analysis

The seedlings of transgenic *A. thaliana* expressing *FgTri101* from each tube with DON or without DON were collected as a single biological replicate, three replicates per treatment. The seedlings were dried with filter paper, flash frozen in liquid nitrogen, then ground using a mortar and pestle. Total RNA was extracted using Trizol reagent (Sigma-Aldrich, St. Louis, MO) combined with the Ambion RNA isolation kit (Hao et al., 2020). RNA was quantified using a spectrophotometer (Nanodrop; Thermo Fisher Scientific, Waltham, MA) and treated with RQ1 RNase-free DNase (Promega Corp. Madison, WI). Library preparation was conducted at Novogene using NEBNext^®^ UltraTM RNA Library Prep Kit for Illumina^®^ (New England Biolabs, Ipswich, MA) following manufacturer’s recommendations. Briefly, the poly-A containing mRNA was purified from about 1 µg total RNA, and fragmented. First strand cDNA was synthesized using random hexamer primers and reverse transcriptase. Subsequently, second stranded cDNA was synthesized using DNA Polymerase I and RNase H. The double stranded cDNA fragments were end-repaired and ligated with adaptors. cDNA fragments of 150~200 bp were size-selected, then amplified with PCR to develop the library. The library quality was assessed on the Agilent Bioanalyzer 2100 system and then used to generate reads using the Illumina HiSeq 2000 system. The raw sequence reads were trimmed and aligned to the *A. thaliana* reference genome (Arabidopsis Genome, 2000). Differentially expressed genes (DEGs) analysis between samples treated with DON and non-treated controls were performed using the EBseq package (Anders and Huber, 2010). DEGs are defined as Fold change >2 between two groups. Gene Ontology (GO) enrichment and pathway enrichment analysis of DEGs were performed to detect significant pathway changes. Focus was given to the genes that encode transporters.

### Gene expression validation by RT-qPCR

Reverse transcriptase-quantitative polymerase chain reaction (RT-qPCR) was conducted to validate the RNA sequencing results for a subset of selected genes. Primers for each gene are listed in **Supplementary Table S1**. cDNA was synthesized using the same RNA used for RNA-seq and RT-PCR reactions were conducted as described (Hao et al., 2019). Briefly, after no DNA contamination in the samples was confirmed, 1 µg RNA was used for cDNA synthesis. Following cDNA synthesis, qPCR was performed on a Bio-Rad CFX96 RealTime System (Bio-Rad Laboratories). The *A. thaliana* gene elongation factor 1-alpha (*EF1α*) was used as an internal control to normalize the expression values. Gene expression was calculated by 2^-ΔΔCt^ method using CFX Manager software (Bio-Rad) (Livak and Schmittgen, 2001).

### Transformation of *A. thaliana Atdtx1* mutant with *FgTRI101*

*Arabidopsis thaliana* (ecotype Columbia, Col-0) transporter mutants, *Atdtx1*(CS923578, polymorphism SALK_064435), *Atdtx4* (SALK_142350C) and *Atabcb4* (SALK_063720C), were obtained from Arabidopsis Biological Resource Center (ABRC, the Ohio State University, OH). *Atdtx1* mutant was confirmed by PCR using a set of primers, *AtDTX1*-LP and RP (**Supplementary Table S1**) that flank T-DNA insertion. *AtdtX3* mutant was confirmed using primers *AtDTX1-*ORF5´ and -ORF3´ (**Supplementary Table S1**). *Atabc4* mutant (pgb4-1, SALK_063720) was confirmed as a homologous mutant by a previous study (Terasaka et al., 2005). All three mutant lines were grown in a growth chamber as described above for seed propagation. The collected seeds were tested for their resistance to DON and ability to transport DON and 3-ADON. Based on the obtained data, *A. thaliana Atdtx1* mutant line was transformed using the construct pBinARS/plus-*FgTRI101* driven by a double 35S (D35S) promoter generated in our previous study (Hao et al., 2021). Transgenic plants were selected on MS media containing 50 mg/L kanamycin. The kanamycin resistant plants were transferred to soil and cultivated in the growth chamber as described above.

### Molecular analysis of transgenic *A. thaliana*

Genomic DNA was isolated from 2- to 3-week-old leaves of kanamycin resistant *A. thaliana* plants using ZR Fungal/Bacterial DNA Miniprep Kit (Zymo Research, Boston, MA). DNA concentration was determined using a spectrophotometer (NanoDrop 2000, Thermofisher Scientific, Waltham, MA) and DNA was used for PCR amplification with the primers FgTRI101-ORF5´ and FgTRI101-ORF3´ (**Supplementary Table S1**).

Total RNA was isolated from leaves of the transgenic *A. thaliana Atdtx1* plants containing *FgTRI101* as described above. The absence of genomic DNA contamination was verified. cDNA synthesis and qPCR were performed as described above. Gene expression level was calculated with the 2^-ΔΔCt^ method (Livak and Schmittgen, 2001) relative to the transgenic plant with the lowest expression level (Tri101-6), which was set as 1. The qPCR reactions were set up in triplicate for each transgenic event and repeated three times with similar results.

The homozygous T2 seeds from transgenic lines were verified by progeny tests on MS media containing 50 mg/L kanamycin. The *FgTRI101* copy number was estimated by qPCR in hemizygous T1 lines using two sets of primers, *FgTRI101*-RT-F/R, *At4HPPD*-F/R (**Supplementary Table S1**) as described (Hao et al., 2021).

### Root resistance assay to DON

Root resistance assays were conducted as described (Hao et al., 2021). Briefly, seeds of *Atdtx1*, *Atdtx3* and *Atabcb4* mutants, T_2_ seeds of the transgenic plants expressing *FgTRI101* in Col-0 background, Tri101-8, and *Atdtx1* mutant background, lines Tri101/*Atdtx1–*2 and-7, along with Col-0 and *Atdtx1* seeds, were surface disinfected as described above. Surface-sterilized seeds were sown on MS medium supplemented with 2, or 10 mg/L DON in square vertical plates (Greiner bio-one North America Inc. Monroe, NC). A total of ten seeds were placed on each agar plate. Each line was tested with three plates. The root length was measured at 17 days after germination.

### DON and 3-ADON transport in *A. thaliana* and transporter mutants

The seeds of *A. thaliana* Col-0 and three transporter mutants, *Atdtx1*, *Atdtx3* and *Atabcb4* were surface disinfected and cultured on MS medium as described above. After about 10 days, 16 to 18 seedlings were each transferred to a 50 mL tube containing 5 mL of half-strength MS liquid medium plus 1% sucrose. The medium was removed after 3–4 days, and fresh medium (5mL) containing 50 mg/L DON or 3-ADON was added. The seedlings and culture media were collected after 24 h incubation. Both seedling samples and culture media were extracted and analyzed for toxins. The fresh weight of seedlings was measured for toxin calculations. Each line was replicated three to four times.

### DON conversion and 3-ADON excretion

T_2_ seeds of the transgenic *A. thaliana FgTRI101–*8 and Tri101/*Atdtx*1–2 and-7, along with Col-0 and *Atdtx1* seeds were surface disinfected, cultured on MS medium, challenged by 50 mg/L DON for 24 h as described above. The fresh weight of seedlings was measured. Both seedling samples and culture media were extracted and analyzed for toxins. Each transgenic line and col-0 were replicated three to four times.

### Mycotoxin extraction and quantification

Mycotoxins were extracted and measured as described (Hao et al., 2021). Briefly, *A. thaliana* seedlings were extracted with 10 ml of acetonitrile: water (86: 14), and 9 mL of extract was dried under a stream of air (Hao et al., 2019). For liquid media, 1.4 mL aliquots were combined with 8.6 mL acetonitrile and the mixture was dried under a stream of air.

Trimethylsilyl (TMS) derivatives were prepared by adding 100 µL of a 100:1 freshly prepared mixture of N-trimethylsilylimadazole/trimethylchlorosilane (Sigma-Aldrich, St. Louis MO) to the dried extract. After 30 min, 900 µL isooctane was added to the reaction mixture followed by 1 mL water. The organic layer was transferred to 2 mL autosampler vial for GC-MS analysis. TMS derivatives of purified DON and 3-ADON, were similarly prepared and used to construct standard curves for quantification.

GC-MS analyses were performed on an Agilent 7890 gas chromatograph fitted with a HP-5MS column (30 m, 0.25 mm, 0.25 µm) with splitless injection and a 5977-mass detector. Samples were analyzed in both scan and selective ion monitoring (SIM) mode. Under these conditions, the TMS ether of DON was detected at 6.14 min (m/z 512, 422, 392, 295, 259, 235 ions), 3-ADON at 6.60 min (m/z 482, 467, 392, 377, 235, 193, 181 ions). Mass Hunter Software with a NIST11 library was used to identify additional peaks in the chromatograms. Toxin content was determined using standard curves. For plant seedling samples, toxin content was divided by seedling fresh weight and compared. For liquid samples, toxin was calculated by volumes.

### Statistical analysis

All statistical analyses were performed using JMP 17 software. The data were analyzed by one-way ANOVA and the means were compared by Tukey honestly significant difference (HSD) test.

## Results

### Identification of *A. thaliana* transporter candidates upregulated by DON/3-ADON

**Figure 1:**
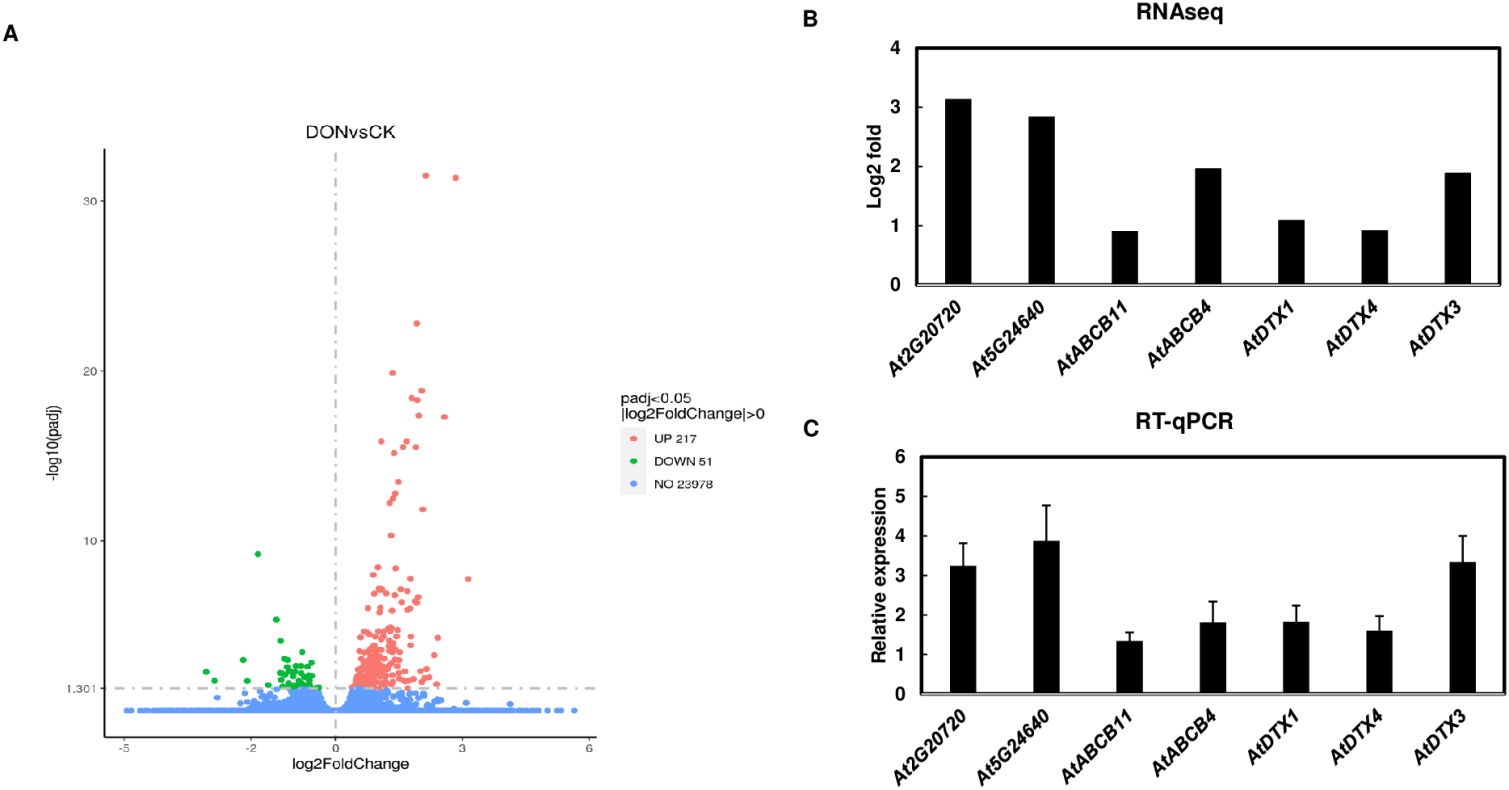
Identification of transporter candidates by RNA seq analyses. **(A)**, Volcano plots for the differentially expressed genes in *Arabidopsis thaliana* transgenic plants expressing *FgTRI101* treated with DON for 24 h compared to controls. Three biological replicates were conducted for each treatment. **(B)**, Selected genes upregulated by DON treatment in RNA seq analysis. **(C)**, Confirmation of the selected gene induction by qPCR. The expression of the selected genes was normalized to the expression of *A. thaliana* elongation factor 1-alpha (*EF1α*). The relative gene expression was calculated using the values of DON treated samples versus the mock treated control. Bars represent the means from three biological replicates and standard deviation.

To identify transporters that are involved in 3-ADON efflux, transgenic *A. thaliana* seedlings expressing *FgTRI101* were treated with 50 µg/mL DON for 24 h. Transcriptomic analyses identified 268 differentially expressed genes (DEG) in DON-treated samples vs. controls and the complete list of DEG is in **Supplementary Table S2**. Out of 268 DEG, 217 A*. thaliana* genes were upregulated, and 51 genes were downregulated by DON or 3-ADON exposure (FgTri101 can convert DON to 3-ADON) (**Figure 1A**). Pathway enrichment analysis revealed a significant response to nitrogen and organonitrogen compounds in DON-treated seedlings than in the control. Genes associated with chitin and bacteria pathways were also enriched (**Supplementary Figure S1**). As expected, UGT-glucosyltransferase activities were upregulated as well (**Supplementary Figure S1**, **Supplementary Table S2**).

The goal of this study was to identify the transporters that export 3-ADON out of plant cells, therefore we focused on transporters that were up regulated in *A. thaliana* FgTri101 seedlings treated with DON. In total, 8 transporter genes were upregulated in the transcriptomic data, including 5 DTXs and 3 ABC transporters (highlighted in yellow in **Supplementary Table S2**). After comparison, three transporter candidates were selected for investigation, including the DTX transporters *AtDTX1*(*At2g04040*) and *AtDTX3*(*At2g04050*), and the ABC transporter *AtABCB4*(*At2g47000*). Both *AtDTX1* gene and *AtDTX3* gene encode 476 amino acid (aa) proteins, and they share 83.4% identity. Both proteins possess a putative transmembrane domain with 12 alpha-helices. In addition, *AtDTX1* is characterized as an efflux carrier for plant derived alkaloids, heavy metals, antibiotics and other toxic compounds (Li et al., 2002). *AtABCB4* encodes a 1286 aa protein that functions as a root-localized auxin influx/efflux transporter and regulates auxin levels in *A. thaliana* roots (Kubes et al., 2012).

To validate RNA-seq data, we confirmed that five transporter genes and two other highly induced genes in RNA seq analysis, *At2g20720* encoding a pentatricopeptide-repeat protein and *At5g24640* that responds to hydrogen peroxide, were induced by DON in RT-qPCR assays (**Figures 1B, C**).

### *AtDTX1* affects 3-ADON transport

Since *Atabc4* mutant (pgb4-1) was confirmed to lack the expected PCR product, gene and protein expression (Terasaka et al., 2005), two selected transporter mutants, *Atdtx1*and *Atdtx3*, were examined and confirmed by PCR in the current study. *Atdtx1* mutant has a T-DNA insertion located at 231 bp after the start codon (**Supplementary Figure S2A**). PCR amplification showed the absence of a PCR product in the *Atdtx1* mutant using the primers flanking T-DNA insertion, whereas the expected PCR band was present in the Col-0 control line (**Supplementary Figure S2B**). *Atdtx3* mutant has a T-DNA insertion in the first exon (**Supplementary Figure S2A**). No PCR product was observed in the *Atdtx3* mutant as well using the primers to amplify *AtDTX3* open reading frame (**Supplementary Figure S2B**). Then, the mutants were tested for their resistance to DON by germinating and growing on medium containing DON. When the mutants were grown on MS medium containing 2 mg/L DON, all three mutants grew as well as Col-0 and the FgTri101 transgenic line. However, when the mutants were placed on MS medium containing 10 mg/L DON, only the FgTri101 transgenic line had root growth, as we previously reported (Hao et al., 2021). No differences in root growth were observed between the transporter mutants and wildtype Col-0 when challenged with 2 mg/L or 10 mg/L DON (**Supplementary Figure S3**). These observations indicate that a single disruption of these transporter candidates does not affect DON toxicity on *A. thaliana* germination and growth.

**Figure 2:**
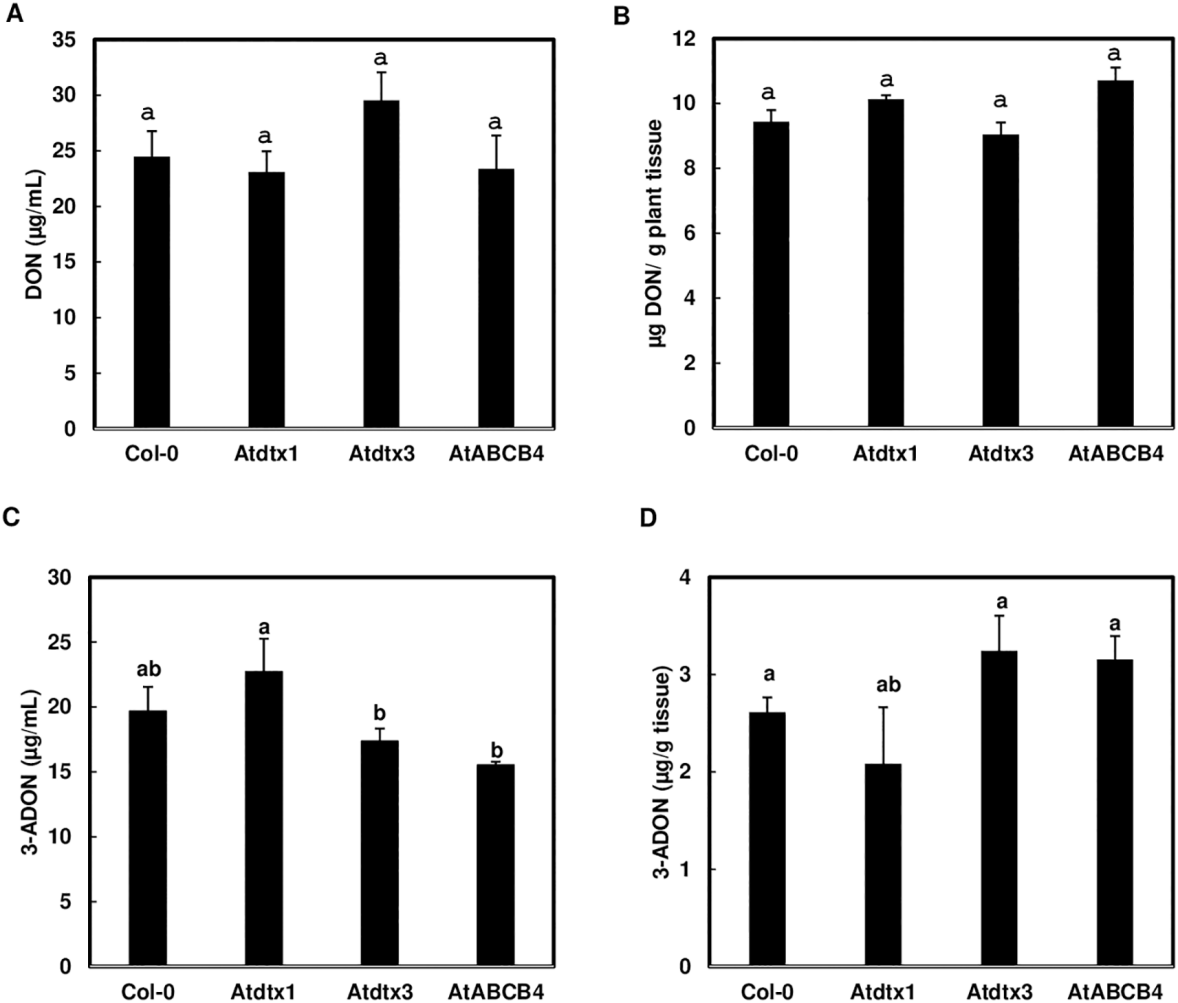
*AtDTX1* affects 3-ADON but not DON uptake. The seedlings of transporter mutants, *Atdtx1*, *Atdtx3*, *AtABCB4* and Col-0 control, were treated with 50 mg/L DON or 3-ADON for 24 h in half Murashige and Skoog (MS) liquid media. **(A)**, DON contents in media; **(B)**, DON contents in seedlings; **(C)**, 3-ADON contents in media; **(D)**, 3-ADON contents in seedlings. Toxins were extracted from seedlings and media separately for comparison. Bars with different letters indicate statistically significant differences.

To determine whether the three transporter candidates are involved in toxin movement, we examined whether disruption of the three *A. thaliana* transporters affects toxin accumulation in liquid media and seedlings. After *A. thaliana* seedlings of Col-0 and three transporter mutants were challenged by 50 mg/L DON, similar levels of DON were observed in seedlings of Col-0 and transporter mutants and within the media (**Figures 2A, B**), suggesting that none of the three transporters affect DON transport alone. When the *A. thaliana* seedlings were challenged by 50 mg/L 3-ADON, a significantly higher level of 3-ADON was found in the media in which *Atdtx1* mutant seedlings were grown, compared to the growth media of *Atdtx3* and *Atabcb4* mutant seedlings (**Figure 2C**), suggesting *AtDTX1* may be involved in 3-ADON transport. However, the accumulation of 3-ADON in Col-0 and transporter mutant seedlings was not significantly different (**Figure 2D**).

### Characterization of transgenic *A. thaliana Atdtx1* mutant expressing *FgTRI101*

**Figure 3:**
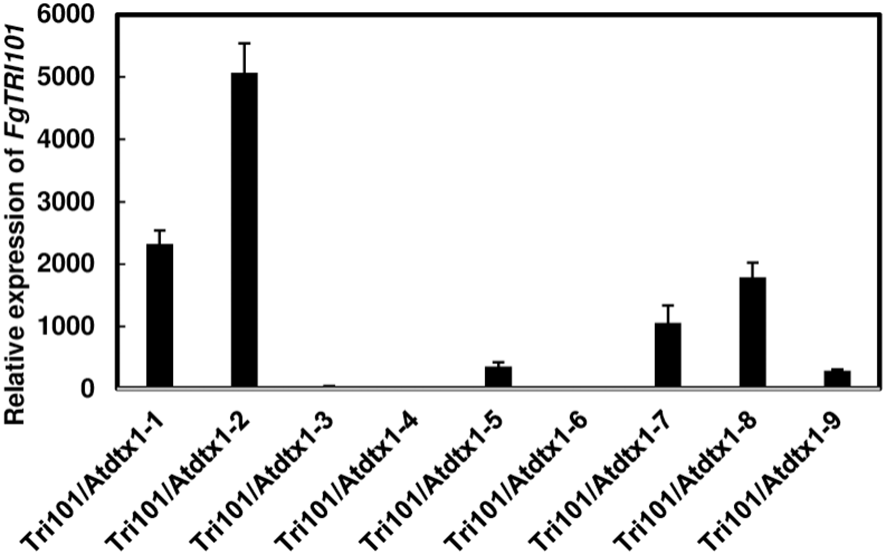
Expression of *FgTRI101* in different transgenic *Arabidopsis thaliana Atdtx1* plants. The expression of *FgTRI101* was normalized to the expression of *A. thaliana* elongation factor 1-alpha (*EF1α*). The relative gene expression is calculated from the 2^-ΔΔCt^ values of a sample versus *A. thaliana* Tri101/*Atdtx1*-6, which had the lowest expression among the tested plants. Each sample was run with three technical replicates in one plate. Bars represent the means from three biological replicates and their standard deviations.

To determine if *AtDTX1* affects 3-ADON efflux, we introduced the *FgTRI101* construct to *Atdtx1* mutant by floral dip transformation. A total of 9 transgenic *Atdtx1* plants were resistant to kanamycin. The presence of *FgTRI101* in *Atdtx1* transgenic plants was confirmed by PCR (**Supplementary Figure S4**). Gene expression analysis by RT-qPCR showed that four transgenic plants, Tri101/*Atdtx1*-1, 2, 7, and 8, had relatively higher expression of *AtDTX1* than the other tested lines (**Figure 3**). Among them, qPCR analysis showed that transgenic lines, Tri101/*Atdtx1*-2, -7, and -8 had a single insertion (**Supplementary Table S3**). Therefore, we selected transgenic lines Tri101/*Atdtx*1–2 and 7, propagated to reach the T2 homozygous generation for further analysis.

### *Atdtx1* lines expressing *FgTRI101* have moderate DON resistance

**Figure 4:**
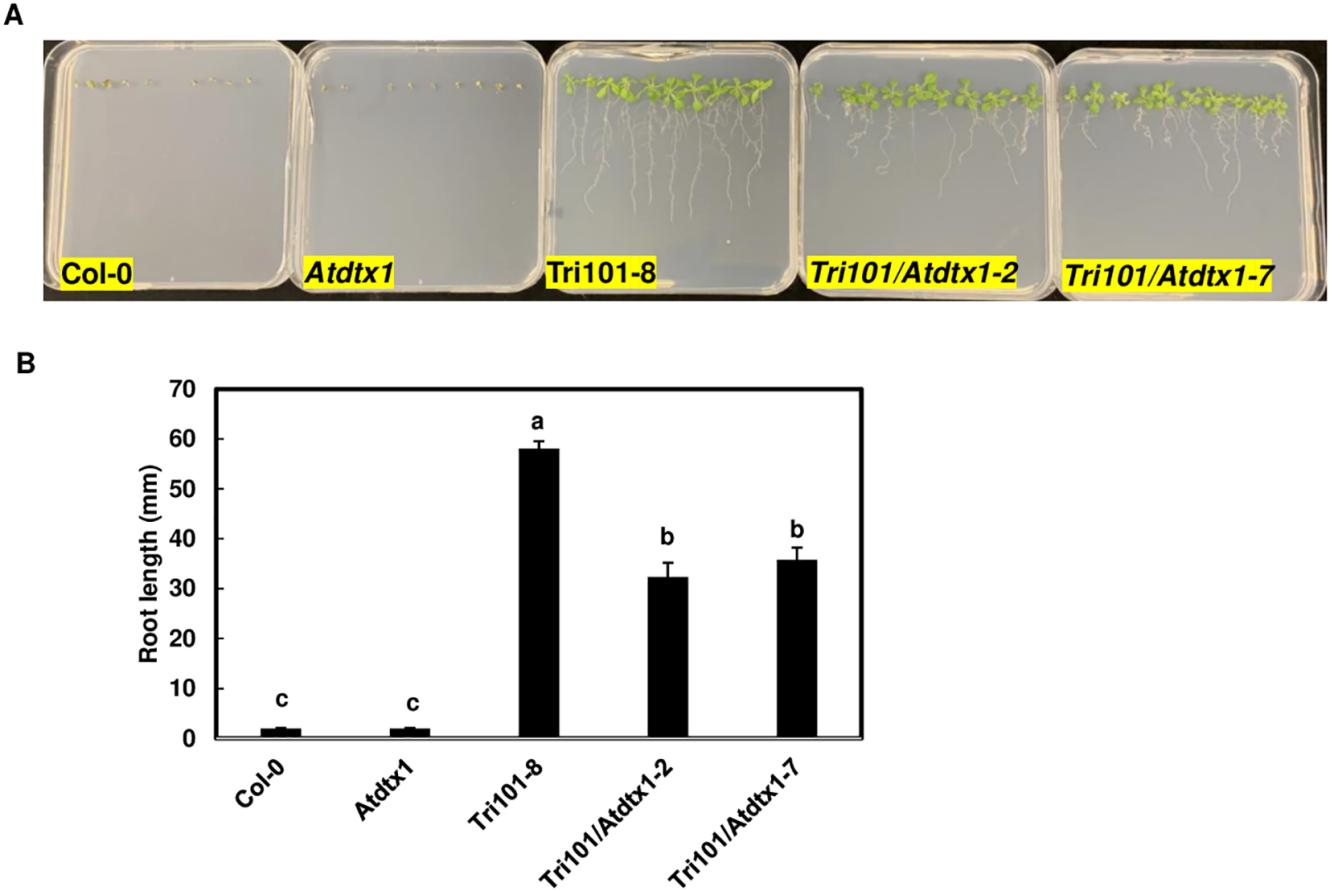
Comparison of growth and toxin accumulation of *Arabidopsis thaliana Atdtx1*mutant line and wildtype expressing *FgTRI101* on Murashige and Skoog (MS) medium containing 10 mg/L DON. **(A)**, Wild-type Col-0, Col-0 expressing *FgTRI101*, *Atdtx1* mutant, and *Atdtx1* lines (FgTri101-2, and 7) expressing *FgTRI101*. The photographs were taken after a two-week period incubation. **(B)**, Root length comparison of different *A. thaliana* seedlings. Root lengths were measured at 14 days. Each line contained 25 to 30 seedlings. Bars with different letters indicate statistically significant differences at p<0.05.

To assess if disruption of *AtDTX1* affects root resistance to DON, we compared root growth of different lines in MS media containing 10 mg/L DON. These lines included *A. thaliana* Col-0 and Col-0 expressing *FgTRI101*, *Atdtx1* mutant and *Atdtx1* mutant expressing *FgTRI101*, lines 2 and 7. As expected, Col-0 and the *Atdtx1* mutant lines germinated but had short roots and yellow cotyledons. Col-0 expressing *FgTRI101* had the longest roots (**Figure 4A**). Statistical analyses showed that the *Atdtx1* lines expressing *FgTRI101*, Tri101/*Atdtx1–*2 and 7, had significantly shorter roots than Col-0 *FgTri101* transgenic plants (**Figure 4B**). These data suggest that *AtDTX1* reduces the efficacy of FgTri101-conferred resistance to DON in *A. thaliana*, possibly by reducing converted 3-ADON excretion in *Atdtx1* lines.

### *AtDTX1* is involved in 3-ADON efflux

**Figure 5:**
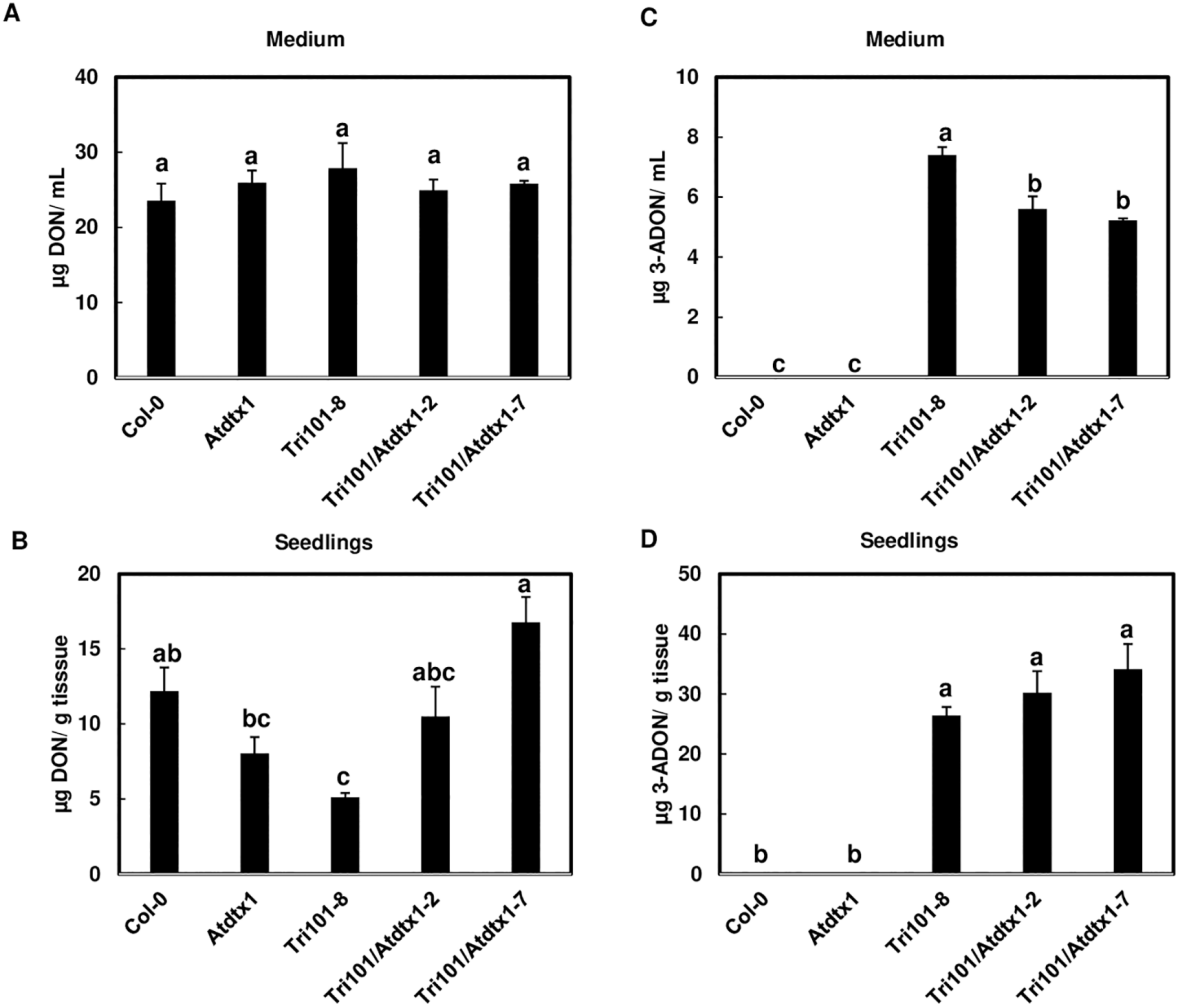
*AtDTX1* is involved in 3-ADON excretion. DON and 3-ADON levels in plants and media after different *A. thaliana* lines, including transporter mutants, Tri101/*Atdtx1-2*, Tri101/*Atdtx1-7*, Tri101-8, *Atdtx1* and Col-0, were grown for 2 days. **(A)**, DON levels in the media after different *A. thaliana* lines treated with 50 mg/L DON for 2 days. **(B)**, DON levels in *A. thaliana* seedlings after treatment with 50 mg/L DON for 24 h. **(C)**, 3-ADON levels present in the media after *A. thaliana* seedlings expressing *FgTRI101* convert DON to 3-ADON. **(D)**, 3-ADON levels converted from DON in *A. thaliana* seedlings expressing *FgTRI101*. Bars with different letters indicate statistically significant differences at p<0.05.

To confirm *AtDTX1* is involved in 3-ADON excretion, we compared 3-ADON levels in liquid half strength MS medium that was used to grow Tri101/*Atdtx1* transgenic seedlings, Col-0, *Atdtx1* and Tri101controls. When DON was added into the media, DON was detected in all media without a significant difference (**Figure 5A**). In seedlings, DON was significantly lower in Tri101 transgenic line compared to the other lines (**Figure 5B**), due to *FgTRI101* converting DON to 3-ADON in the transgenic seedlings expressing *FgTRI101*. As expected, no 3-ADON was detected in Col-0 or *Atdtx1* mutant seedlings or in the culturing media, due to non-Tri101 seedlings lacking the ability to convert DON to 3-ADON (**Figures 5C, D**). Significantly less 3-ADON was detected in the media in which the Tri101/A*tdtx1* lines were grown than that of the Tri101 line (**Figure 5C**), indicating that Tri101/*Atdtx1* lines is less efficient in 3-ADON efflux than the Tri101 line. However, the difference in 3-ADON level was not significantly different in Tri101/*Atdtx1* or Tri101 seedlings (**Figure 5D**), which may be due to 3-ADON conversion to other forms of metabolites or the presence of an additional transporter with functional redundancy. Overall, these data confirm that *AtDTX1* is involved in 3-ADON efflux but also suggested that another transporter may be associated with 3-ADON efflux.

## Discussion

Transporters play an important role in protecting fungi from mycotoxins. In some *Fusarium* species, the Tri12 transporter exports trichothecene analogs for fungal protection (Alexander et al., 2009). Similarly, in *Saccharomyces cerevisiae* a pleiotropic drug resistance (PDR)-like ABC transporter, PDR5 (Balzi et al., 1994), exports DON from the cytoplasm into the extracellular space (Gunter et al., 2016). Plant transporters also play a critical role in sequestration or excretion of mycotoxins. A previous study identified two vacuolar membrane-localized transporters in wheat, TaABCC3.1 and TaABCC3.2, which are induced by DON and associated with DON sequestration (Walter et al., 2015). Furthermore, TaABCC3.1 transporter was shown to contribute to wheat resistance to FHB (Walter et al., 2015). It has been shown that trichothecenes, such as DON, can cause plant cell death, tissue chlorosis, root growth inhibition and wheat spike bleaching during fungal infection (Desmond et al., 2008; Wipfler et al., 2019; Hao et al., 2021). However, little is known about whether plant cells take up trichothecenes passively, actively or both. Here, we identified the first *A. thaliana* transporter, *AtDTX1* involved in trichothecene 3-ADON export.

After we showed that transgenic *A. thaliana* expressing *FgTRI101* can convert DON to 3-ADON and excrete 3-ADON out of plant cells for plant protection (Hao et al., 2021), we conducted experiments to identify DON or 3-ADON transporters. Using transcriptomic analyses, three potential transporters for trichothecene transport were identified. Surprisingly, none of these transporters significantly affected DON levels in seedlings or the media used to culture them compared to Col-0 controls, suggesting that these three transporters are not involved in DON transport or function redundantly (**Figure 2A**). We speculate that multiple transporters are involved in DON transport or DON as a small molecule is able to diffuse in/out of plant cells. Trichothecenes have a core structure, 12, 13-epoxytrichothec-9-ene, and they are classified into different groups according to different side chains attached to the core structure (McCormick et al., 2011). It is possible that there are specific transporters for different trichothecene analogs because the *Atdtx1* mutant did not significantly affect DON transport. Further investigations are needed to clarify these possibilities.

In the transcriptomic analysis of Tri101 *A. thaliana* seedlings, many differently expressed genes were identified after DON treatment. Several UDP-glycosyltransferases (UGTs) were upregulated in *A. thaliana* seedlings treated with DON. When one of them, *A. thaliana* UGT73C5 (DOGT1) was expressed in *S. cerevisiae*, it conferred DON resistance by converting DON to the less toxic DON-3-O-glucoside (Poppenberger et al., 2003). Although *A. thaliana* over-expressing AtUGt73C5 had increased DON resistance, the transgenic plants were dwarfs due to the formation of brassinosteroid-glucosides (Poppenberger et al., 2003). In this study, as expected, the expression of *A. thaliana* UGTs was induced by exposure to DON, to reduce DON toxicity (**Supplementary Table S2**). Many genes associated with plant defense responses were also up-regulated in seedlings treated with DON, such as ethylene-responsive transcription factor CRF5 and PAMP-induced secreted peptide 1 (**Supplementary Table S2**). Although DON is not a virulence factor in *A. thaliana*, it can cause plant cell death, leaf chlorosis, and inhibit root growth (Hao et al., 2021). Therefore, it is plausible that plant defense genes were enriched when *A. thaliana* seedlings were treated with DON.

Transgenic *A. thaliana* mutant *Atdtx1* plants expressing *FgTRI101* displayed some resistance to DON but had significantly shorter roots than *A. thaliana* Col-0 expressing *FgTRI101* (**Figure 4**). DON inhibits *A. thaliana* root growth and causes dwarfism (Masuda et al., 2007; Hao et al., 2021). In contrast, 3-ADON is less toxic to the roots of *A. thaliana* and rice (Ohsato et al., 2007; Hao et al., 2021). When challenged with DON in liquid culture assays, significantly less 3-ADON was detected in the growth media for *Atdtx1* seedlings expressing *FgTRI101* than Col-0 expressing *FgTRI101* (**Figure 5C**). *AtDTX1* has been reported to localize in plasma membranes and to export toxins out of *A. thaliana* cells (Li et al., 2002). Taken together, these studies demonstrated that *AtDTX1* is an efflux carrier for 3-ADON export. A prior study showed that the wheat transporter TaABCC3.1 contributed to FHB resistance (Walter et al., 2015). Although 3-ADON is less toxic to plants than DON, 3-ADON and DON have similar toxicity on animals (Broekaert et al., 2017). In contaminated grains, different trichothecene analogs often co-exist. 3-ADON accounts for approximately 20% of the total DON content in contaminated grains. A study also found that the naturally contaminated triticale kernels contain about 59% 3-ADON and 30% DON (Perkowski and Kaczmarek, 2002). In recent years, the presence of these acetylated forms has become more prominent because they increase total toxin contents in food and feed (Janaviciene et al., 2018). Future studies to determine whether *AtDTX1* homologs in wheat affect FHB resistance and overexpression of *AtDTX1* can reduce mycotoxin toxicity are required.

In summary, using transcriptomic and trichothecene resistance studies, we identified an *A. thaliana* transporter *AtDTX1* that is involved in 3-ADON transport. Furthermore, we demonstrated that *A. thaliana AtDTX1* participates in 3-ADON efflux by expressing *FgTRI101* in *Atdtx1* mutant background. To the best of our knowledge, this is the first plant transporter identified for trichothecene 3-ADON export. It is our future interest to determine if *AtDTX1* is involved in transport of other mycotoxins. Expression of *AtDTX1* or co-expression of *FgTRI101* and *AtDTX1* in wheat may lead to enhanced FHB resistance and mycotoxin reduction.

## Data Availability

The data presented in the study are deposited at NCBI, accession number PRJNA1259505.

## Supplementary material

Supplementary Figure 1 Pathway enrichment analysis of differentially expressed genes (DEGs) in *FgTRI101 Arabidopsis thaliana* seedlings treated with DON versus untreated controls.

Supplementary Figure 2 Confirmation of *Atdtx1* and *Atdtx3* mutants. **(A)**, Gene structures of *AtDTX1* and *AtDTX3* with T-DNA insertion sites of *Atdtx1* (SALK_064435) and *Atdtx3* (SALK_067653). Shaded rectangles represent exons, and lines represent untranslated regions, including the introns. Triangles indicate T-DNA. ATG, start code, TAA, stop code. LP, RP, ORF5´ and ORF3´ represent primers. **(B)**, PCR amplification of *AtDTX1* and *AtDTX3* in *Arabidopsis* plants and mutants. Genomic DNA was used for PCR. M: DNA marker; Col-0: Wild type control; W: Water as a negative control; *Atdtx1*: and *Atdtx3* mutants. Arrow indicates the size of PCR products.

Supplementary Figure 3 DON has no effect on root growth of three *Arabidopsis thaliana* transporter mutants. *A. thaliana* Col-0 and transporter mutants, *Atdtx1, Atdtx3* and *Atabcb4*, were grown on MS media containing DON at **(A)**, 2 mg/L or **(B)**, 10 mg/L. The photographs were taken after a two-week incubation.

Supplementary Figure 4 PCR amplification of *FgTRI101* gene from transgenic *Arabidopsis thaliana Atdtx1* plants. Genomic DNA was amplified with *FgTRI101* primers ORF5′ and ORF3′. M: DNA marker; N: Water as a negative control; Lanes 1-9: Transgenic plants containing *FgTRI101*. Arrow indicates the size of PCR product.

## References

[B1] Alexander N. J. Proctor R. H. McCormick S. P. (2009). Genes, gene clusters, and biosynthesis of trichothecenes and fumonisins in *Fusarium* . Toxin. Rev. 28, 198–215. doi: 10.1080/15569540903092142

[B2] Anders S. Huber W. (2010). Differential expression analysis for sequence count data. Genome Biol. 11, R106. doi: 10.1186/gb-2010-11-10-r106 20979621 PMC3218662

[B3] Arabidopsis Genome I. (2000). Analysis of the genome sequence of the flowering plant *Arabidopsis thaliana* . Nature 408, 796–815. doi: 10.1038/35048692 11130711

[B4] Armer V. J. Urban M. Ashfield T. Deeks M. J. Hammond-Kosack K. E. (2024). The trichothecene mycotoxin deoxynivalenol facilitates cell-to-cell invasion during wheat-tissue colonization by *Fusarium graminearum* . Mol. Plant Pathol. 25, e13485. doi: 10.1111/mpp.13485 38877764 PMC11178975

[B5] Bai G. H. Desjardins A. E. Plattner R. D. (2002). Deoxynivalenol-nonproducing *Fusarium graminearum* causes initial infection, but does not cause disease spread in wheat spikes. Mycopathologia 153, 91–98. doi: 10.1023/A:1014419323550 12000132

[B6] Balzi E. Wang M. Leterme S. Van Dyck L. Goffeau A. (1994). PDR5, a novel yeast multidrug resistance conferring transporter controlled by the transcription regulator PDR1. J. Biol. Chem. 269, 2206–2214. doi: 10.1016/S0021-9258(17)42155-7 8294477

[B7] Bin-Umer M. A. McLaughlin J. E. Basu D. McCormick S. Tumer N. E. (2011). Trichothecene mycotoxins inhibit mitochondrial translation–implication for the mechanism of toxicity. Toxins. (Basel). 3, 1484–1501. doi: 10.3390/toxins3121484 22295173 PMC3268453

[B8] Broekaert N. Devreese M. van Bergen T. Schauvliege S. De Boevre M. De Saeger S.. (2017). *In vivo* contribution of deoxynivalenol-3-beta-D-glucoside to deoxynivalenol exposure in broiler chickens and pigs: oral bioavailability, hydrolysis and toxicokinetics. Arch. Toxicol. 91, 699–712. doi: 10.1007/s00204-016-1710-2 27100115

[B9] Chen Q. Wang L. Liu D. Ma S. Dai Y. Zhang X.. (2020). Identification and expression of the multidrug and toxic compound extrusion (MATE) gene family in *Capsicum annuum* and *Solanum tuberosum* . Plants (Basel). 9. doi: 10.3390/plants9111448 PMC7716203 33120967

[B10] Desjardins A. E. McCormick S. P. Appell M. (2007). Structure-activity relationships of trichothecene toxins in an *Arabidopsis thaliana* leaf assay. J. Agric. Food Chem. 55, 6487–6492. doi: 10.1021/jf0709193 17630765

[B11] Desmond O. J. Manners J. M. Stephens A. E. Maclean D. J. Schenk P. M. Gardiner D. M.. (2008). The Fusarium mycotoxin deoxynivalenol elicits hydrogen peroxide production, programmed cell death and defence responses in wheat. Mol. Plant Pathol. 9, 435–445. doi: 10.1111/j.1364-3703.2008.00475.x 18705859 PMC6640518

[B12] Dobritzsch M. Lubken T. Eschen-Lippold L. Gorzolka K. Blum E. Matern A.. (2016). MATE transporter-dependent export of hydroxycinnamic acid amides. Plant Cell 28, 583–596. doi: 10.1105/tpc.15.00706 26744218 PMC4790871

[B13] Foroud N. A. Baines D. Gagkaeva T. Y. Thakor N. Badea A. Steiner B.. (2019). Trichothecenes in cereal grains - an update. Toxins. (Basel). 11, 634. doi: 10.3390/toxins11110634 31683661 PMC6891312

[B14] Garreau de Loubresse N. Prokhorova I. Holtkamp W. Rodnina M. V. Yusupova G. Yusupov M. (2014). Structural basis for the inhibition of the eukaryotic ribosome. Nature 513, 517–522. doi: 10.1038/nature13737 25209664

[B15] Gunter A. B. Hermans A. Bosnich W. Johnson D. A. Harris L. J. Gleddie S. (2016). Protein engineering of *Saccharomyces cerevisiae* transporter Pdr5p identifies key residues that impact Fusarium mycotoxin export and resistance to inhibition. Microbiologyopen 5, 979–991. doi: 10.1002/mbo3.2016.5.issue-6 27263049 PMC5221463

[B16] Hao G. Bakker M. G. Kim H. S. (2020). Enhanced resistance to *Fusarium graminearum* in transgenic arabidopsis plants expressing a modified plant thionin. Phytopathology. PHYTO12190447R. 110, 1056–1066. doi: 10.1094/PHYTO-12-19-0447-R 32043419

[B17] Hao G. McCormick S. Tiley H. Usgaard T. (2021). Detoxification and excretion of trichothecenes in transgenic *Arabidopsis thaliana* expressing *Fusarium graminearum* trichothecene 3-O-acetyltransferase. Toxins. (Basel). 13, 320. doi: 10.3390/toxins13050320 33946742 PMC8145220

[B18] Hao G. McCormick S. Vaughan M. M. Naumann T. A. Kim H. S. Proctor R.. (2019). *Fusarium graminearum* arabinanase (Arb93B) enhances wheat head blight susceptibility by suppressing plant immunity. Mol. Plant Microbe Interact. 32, 888–898. doi: 10.1094/MPMI-06-18-0170-R 30759350

[B19] Janaviciene S. Mankeviciene A. Suproniene S. Kochiieru Y. Keriene I. (2018). The prevalence of deoxynivalenol and its derivatives in the spring wheat grain from different agricultural production systems in Lithuania. Food Addit. Contam. Part A Chem. Anal. Control. Expo. Risk Assess. 35, 1179–1188. doi: 10.1080/19440049.2018.1427893 29337657

[B20] Kang J. Park J. Choi H. Burla B. Kretzschmar T. Lee Y.. (2011). Plant ABC transporters. Arabidopsis book (Springer), Vol. 9, e0153. doi: 10.1199/tab.0153 22303277 PMC3268509

[B21] Kubes M. Yang H. Richter G. L. Cheng Y. Mlodzinska E. Wang X.. (2012). The Arabidopsis concentration-dependent influx/efflux transporter ABCB4 regulates cellular auxin levels in the root epidermis. Plant J. 69, 640–654. doi: 10.1111/j.1365-313X.2011.04818.x 21992190

[B22] Li L. He Z. Pandey G. K. Tsuchiya T. Luan S. (2002). Functional cloning and characterization of a plant efflux carrier for multidrug and heavy metal detoxification. J. Biol. Chem. 277, 5360–5368. doi: 10.1074/jbc.M108777200 11739388

[B23] Li X. Shin S. Heinen S. Dill-Macky R. Berthiller F. Nersesian N.. (2015). Transgenic wheat expressing a barley UDP-glucosyltransferase detoxifies deoxynivalenol and provides high levels of resistance to *Fusarium graminearum* . Mol. Plant Microbe Interact. 28, 1237–1246. doi: 10.1094/MPMI-03-15-0062-R 26214711

[B24] Livak K. J. Schmittgen T. D. (2001). Analysis of relative gene expression data using real-time quantitative PCR and the 2(-Delta Delta C(T)) Method. Methods 25, 402–408. doi: 10.1006/meth.2001.1262 11846609

[B25] Lu Y. P. Li Z. S. Drozdowicz Y. M. Hortensteiner S. Martinoia E. Rea P. A. (1998). AtMRP2, an Arabidopsis ATP binding cassette transporter able to transport glutathione S-conjugates and chlorophyll catabolites: functional comparisons with Atmrp1. Plant Cell 10, 267–282. doi: 10.1105/tpc.10.2.267 9490749 PMC143980

[B26] Lu Y. P. Li Z. S. Rea P. A. (1997). AtMRP1 gene of Arabidopsis encodes a glutathione S-conjugate pump: isolation and functional definition of a plant ATP-binding cassette transporter gene. Proc. Natl. Acad. Sci. U. S. A 94, 8243–8248. doi: 10.1073/pnas.94.15.8243 9223346 PMC21588

[B27] Marinova K. Pourcel L. Weder B. Schwarz M. Barron D. Routaboul J. M.. (2007). The Arabidopsis MATE transporter TT12 acts as a vacuolar flavonoid/H+ -antiporter active in proanthocyanidin-accumulating cells of the seed coat. Plant Cell 19, 2023–2038. doi: 10.1105/tpc.106.046029 17601828 PMC1955721

[B28] Masuda D. Ishida M. Yamaguchi K. Yamaguchi I. Kimura M. Nishiuchi T. (2007). Phytotoxic effects of trichothecenes on the growth and morphology of *Arabidopsis thaliana* . J. Exp. Bot. 58, 1617–1626. doi: 10.1093/jxb/erl298 17426057

[B29] McCormick S. P. Stanley A. M. Stover N. A. Alexander N. J. (2011). Trichothecenes: from simple to complex mycotoxins. Toxins. (Basel). 3, 802–814. doi: 10.3390/toxins3070802 22069741 PMC3202860

[B30] Morita M. Shitan N. Sawada K. Van Montagu M. C. Inze D. Rischer H.. (2009). Vacuolar transport of nicotine is mediated by a multidrug and toxic compound extrusion (MATE) transporter in *Nicotiana tabacum* . Proc. Natl. Acad. Sci. U. S. A 106, 2447–2452. doi: 10.1073/pnas.0812512106 19168636 PMC2650162

[B31] Ohsato S. Ochiai-Fukuda T. Nishiuchi T. Takahashi-Ando N. Koizumi S. Hamamoto H.. (2007). Transgenic rice plants expressing trichothecene 3-O-acetyltransferase show resistance to the *Fusarium phytotoxin* deoxynivalenol. Plant Cell Rep. 26, 531–538. doi: 10.1007/s00299-006-0251-1 17031651

[B32] Perkowski J. Kaczmarek Z. (2002). Distribution of deoxynivalenol and 3-acetyldeoxynivalenol in naturally contaminated and *Fusarium culmorum* infected triticale samples. Nahrung 46, 415–419. doi: 10.1002/1521-3803(20021101)46:6<415::AID-FOOD415>3.0.CO;2-0 12577591

[B33] Poppenberger B. Berthiller F. Lucyshyn D. Sieberer T. Schuhmacher R. Krska R.. (2003). Detoxification of the Fusarium mycotoxin deoxynivalenol by a UDP-glucosyltransferase from *Arabidopsis thaliana* . J. Biol. Chem. 278, 47905–47914. doi: 10.1074/jbc.M307552200 12970342

[B34] Proctor R. H. Hohn T. M. McCormick S. P. (1995). Reduced virulence of *Gibberella zeae* caused by disruption of a trichothecene toxin biosynthetic gene. Mol. Plant Microbe Interact. 8, 593–601. doi: 10.1094/MPMI-8-0593 8589414

[B35] Rocha O. Ansari K. Doohan F. M. (2005). Effects of trichothecene mycotoxins on eukaryotic cells: A review. Food Addit. Contam. A 22, 369–378. doi: 10.1080/02652030500058403 16019807

[B36] Terasaka K. Blakeslee J. J. Titapiwatanakun B. Peer W. A. Bandyopadhyay A. Makam S. N.. (2005). PGP4, an ATP binding cassette P-glycoprotein, catalyzes auxin transport in *Arabidopsis thaliana* roots. Plant Cell 17, 2922–2939. doi: 10.1105/tpc.105.035816 16243904 PMC1276020

[B37] Thompson E. P. Wilkins C. Demidchik V. Davies J. M. Glover B. J. (2010). An Arabidopsis flavonoid transporter is required for anther dehiscence and pollen development. J. Exp. Bot. 61, 439–451. doi: 10.1093/jxb/erp312 19995827 PMC2803208

[B38] Walter S. Kahla A. Arunachalam C. Perochon A. Khan M. R. Scofield S. R.. (2015). A wheat ABC transporter contributes to both grain formation and mycotoxin tolerance. J. Exp. Bot. 66, 2583–2593. doi: 10.1093/jxb/erv048 25732534 PMC4986867

[B39] Wang H. Sun S. Ge W. Zhao L. Hou B. Wang K.. (2020). Horizontal gene transfer of Fhb7 from fungus underlies Fusarium head blight resistance in wheat. Science. 368, 6493. doi: 10.1126/science.aba5435 32273397

[B40] Wipfler R. McCormick S. P. Proctor R. Teresi J. Hao G. Ward T.. (2019). Synergistic phytotoxic effects of culmorin and trichothecene mycotoxins. Toxins. (Basel). 11, 555. doi: 10.3390/toxins11100555 31547160 PMC6833022

[B41] Yulfo-Soto G. McCormick S. Chen H. Bai G. Trick H. N. Hao G. (2024). Reduction of Fusarium head blight and trichothecene contamination in transgenic wheat expressing *Fusarium graminearum* trichothecene 3-O-acetyltransferase. Front. Plant Sci. 15, 1389605. doi: 10.3389/fpls.2024.1389605 38650698 PMC11033581

[B42] Zhang H. Zhao F. G. Tang R. J. Yu Y. Song J. Wang Y.. (2017). Two tonoplast MATE proteins function as turgor-regulating chloride channels in Arabidopsis. Proc. Natl. Acad. Sci. U. S. A 114, E2036–E2045. doi: 10.1073/pnas.1616203114 28202726 PMC5347570

