# Supplementary figures and images for: Arabidopsis thaliana detoxification gene AtDTX1 is involved in trichothecene 3-acetyl-deoxynivalenol efflux
Source: Front Plant Sci. 2025 May 19;16:1574367. doi: 10.3389/fpls.2025.1574367 (PMC12127357; doi:10.3389/fpls.2025.1574367)

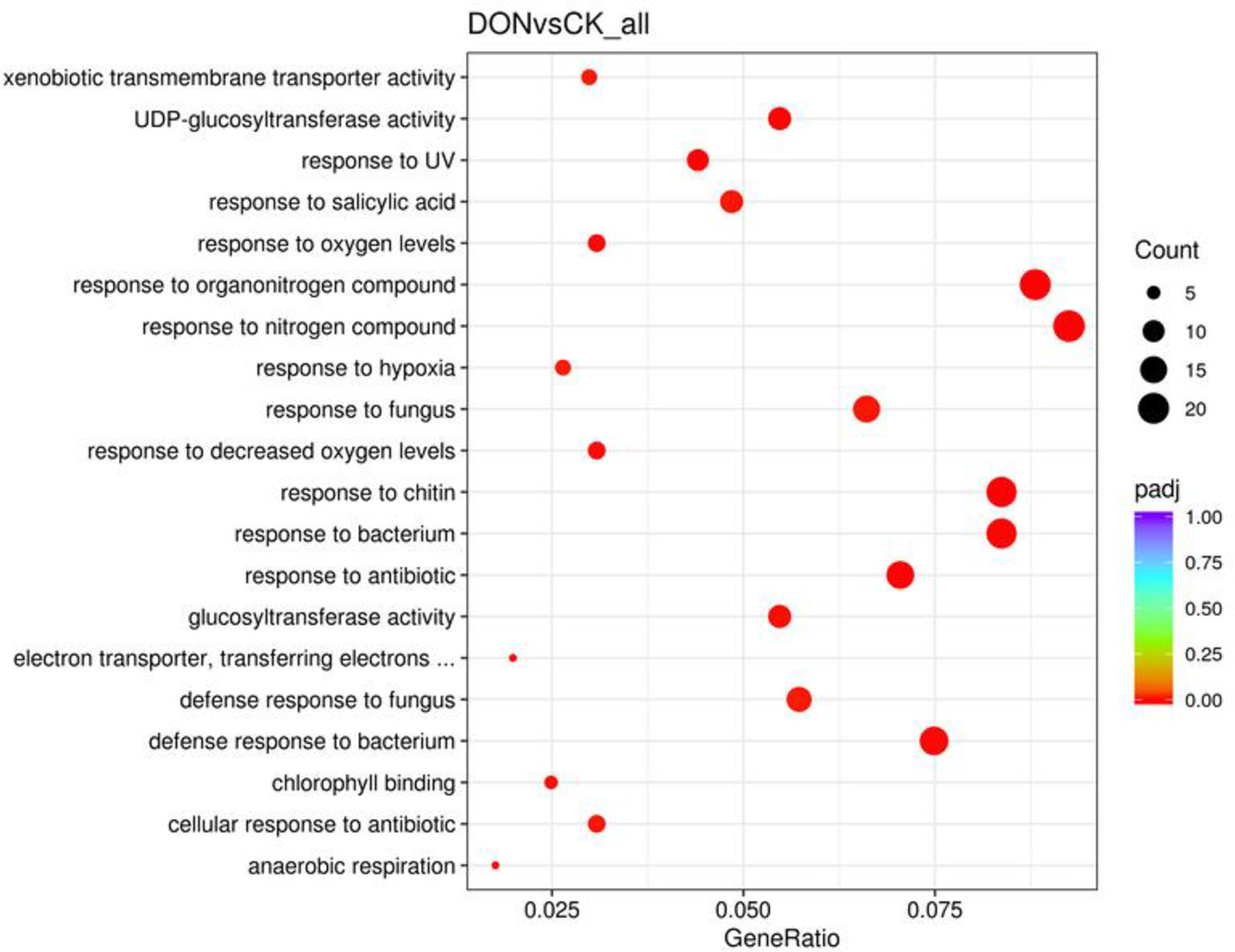

Supplement: Supplementary Figure 1 — Pathway enrichment analysis of differentially expressed genes (DEGs) in FgTRI101 Arabidopsis thaliana seedlings treated with DON versus untreated controls. [file Image1.tif]

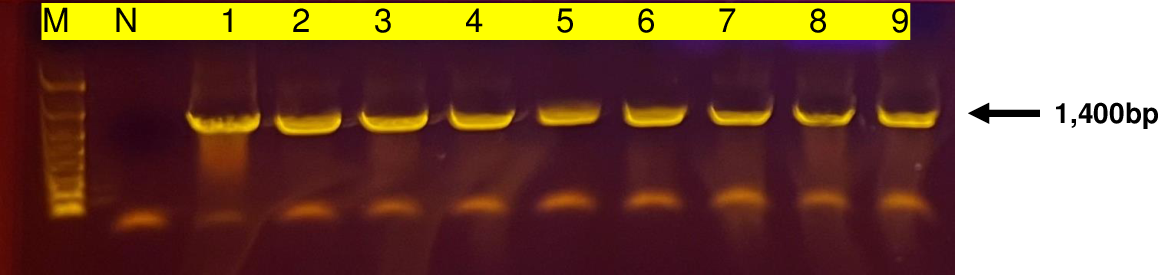

Supplement: Supplementary Figure 2 — Confirmation of Atdtx1 and Atdtx3 mutants. (A), Gene structures of AtDTX1 and AtDTX3 with T-DNA insertion sites of Atdtx1 (SALK_064435) and Atdtx3 (SALK_067653). Shaded rectangles represent exons, and lines represent untranslated regions, including the introns. Triangles indicate T-DNA. ATG, start code, TAA, stop code. LP, RP, ORF5´ and ORF3´ represent primers. (B), PCR amplification of AtDTX1 and AtDTX3 in Arabidopsis plants and mutants. Genomic DNA was used for PCR. M: DNA marker; Col-0: Wild type control; W: Water as a negative control; Atdtx1: and Atdtx3 mutants. Arrow indicates the size of PCR products. [file Image2.tif]

A

*Atdtx1* (SALK\_064435)

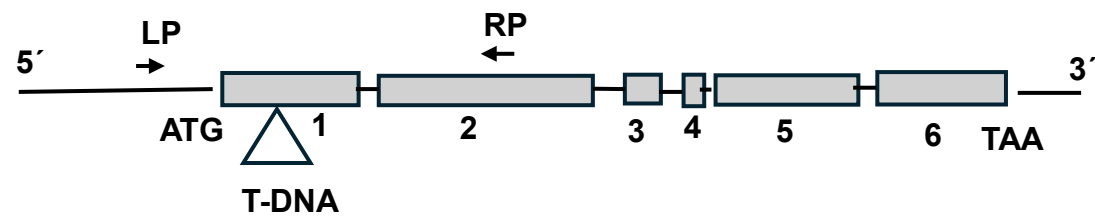

*Atdtx3* (SALK\_142350)

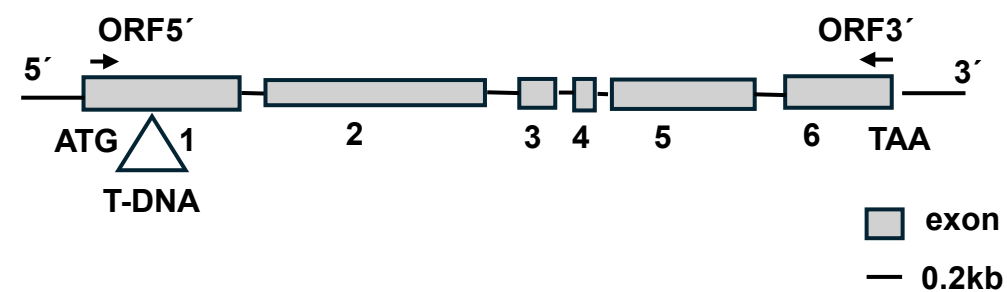

B

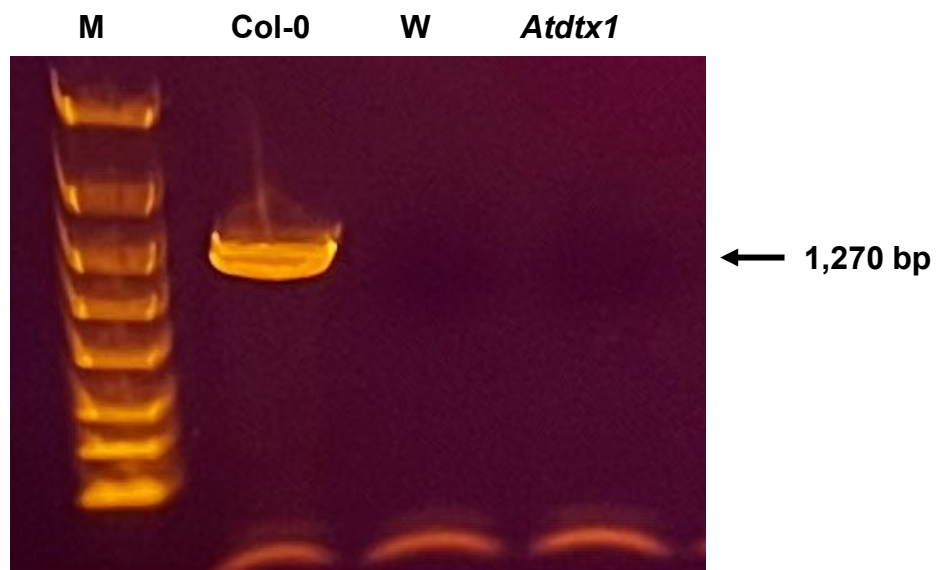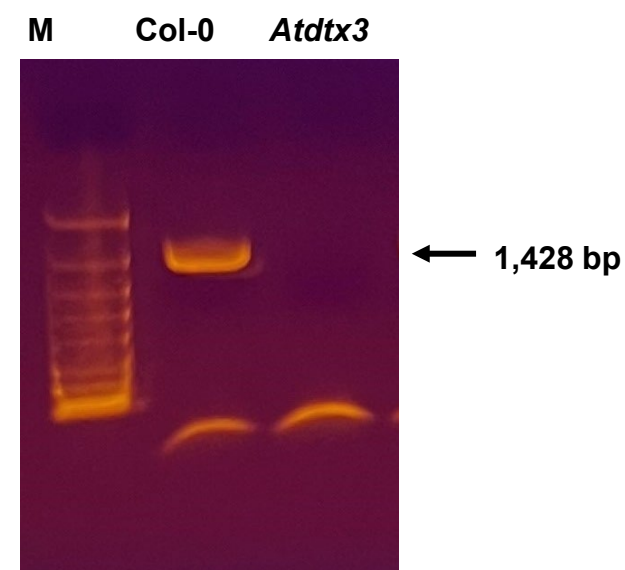

Supplement: Supplementary Figure 3 — DON has no effect on root growth of three Arabidopsis thaliana transporter mutants. A. thaliana Col-0 and transporter mutants, Atdtx1, Atdtx3 and Atabcb4, were grown on MS media containing DON at (A), 2 mg/L or (B), 10 mg/L. The photographs were taken after a two-week incubation. [file DataSheet1.pdf]

**Col-0**

**Tri101**

***Atdtx1***

***Atabcb4***

***Atdtx3***

**A**

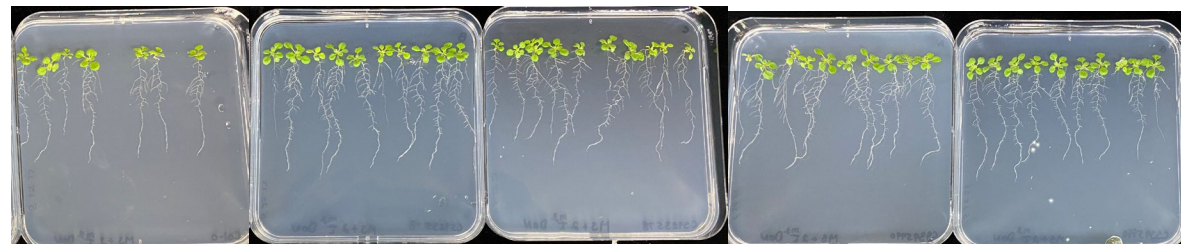

**B**

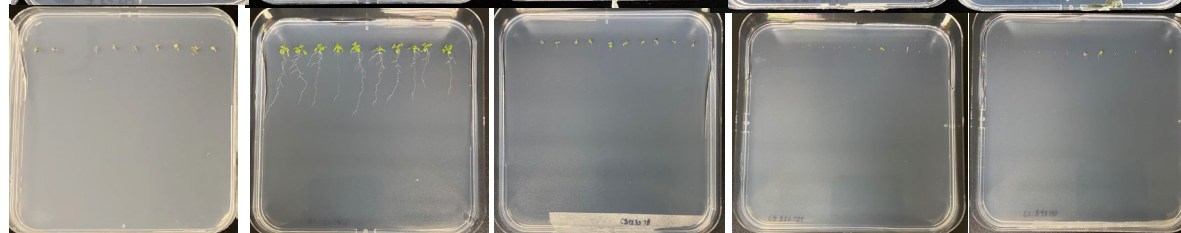

Supplement: Supplementary Figure 4 — PCR amplification of FgTRI101 gene from transgenic Arabidopsis thaliana Atdtx1 plants. Genomic DNA was amplified with FgTRI101 primers ORF5′ and ORF3′. M: DNA marker; N: Water as a negative control; Lanes 1-9: Transgenic plants containing FgTRI101. Arrow indicates the size of PCR product. [file DataSheet2.pdf]
